# Intact parathyroid hormone levels localize causative glands in persistent or recurrent renal hyperparathyroidism: A retrospective cohort study

**DOI:** 10.1371/journal.pone.0248366

**Published:** 2021-04-01

**Authors:** Takahisa Hiramitsu, Toshihide Tomosugi, Manabu Okada, Kenta Futamura, Norihiko Goto, Shunji Narumi, Yoshihiko Watarai, Yoshihiro Tominaga, Toshihiro Ichimori

**Affiliations:** Department of Transplant and Endocrine Surgery, Nagoya Daini Red Cross Hospital, Nagoya, Aichi, Japan; University of Mississippi Medical Center, UNITED STATES

## Abstract

Persistent or recurrent renal hyperparathyroidism may occur after total parathyroidectomy and transcervical thymectomy with forearm autograft under continuous stimulation due to uremia. Parathyroid hormone (PTH) levels may reflect persistent or recurrent renal hyperparathyroidism because of the enlarged autografted parathyroid glands in the forearm or remnant parathyroid glands in the neck or mediastinum. Detailed imaging requires predictive localization of causative parathyroid glands. Casanova and simplified Casanova tests may be convenient. However, these methods require avascularization of the autografted forearm for >10 min with a tourniquet or Esmarch. The heavy pressure during avascularization can be incredibly painful and result in nerve damage. An easier method that minimizes the burden on patients in addition to predicting the localization of causative parathyroid glands was developed in this study. Ninety patients who underwent successful re-parathyroidectomy for persistent or recurrent renal hyperparathyroidism after parathyroidectomy between January 2000 and July 2019 were classified according to the localization of causative parathyroid glands (63 and 27 patients in the autografted forearm and the neck or mediastinum groups, respectively). Preoperatively, intact PTH levels were measured from bilateral forearm blood samples following a 5-min avascularization of the autografted forearm. Cutoff values of the intact PTH ratio (intact PTH level obtained from the non-autografted forearm before re-parathyroidectomy/intact PTH level obtained from the autografted forearm before re-parathyroidectomy) were investigated with receiver operating characteristic curves to localize the causative parathyroid glands. Intact PTH ratios of <0.310 with an area under the curve (AUC) of 0.913 (95% confidence interval [CI]: 0.856–0.970; P < 0.001) and >0.859 with an AUC 0.744 (95% CI: 0.587–0.901; P = 0.013) could predict causative parathyroid glands in the autografted forearm and the neck or mediastinum with diagnostic accuracies of 81.1% and 83.3%, respectively. Therefore, we propose that the intact PTH ratio is useful for predicting the localization of causative parathyroid glands for re-parathyroidectomy.

## Introduction

Recurrent or persistent renal hyperparathyroidism (rHPT) may develop in patients with end-stage renal disease (ESRD) after total parathyroidectomy (PTx) and transcervical thymectomy with forearm autograft under continuous stimulation due to uremia [1]. Decreased renal excretion of phosphorus and activated vitamin D deficiency cause hyperphosphatemia and hypocalcemia, respectively, and stimulate the remnant or autografted parathyroid glands (PTGs). For the localization of causative PTGs, the efficacies of various imaging studies have been reported [2–8]. Intact parathyroid hormone (PTH) levels are routinely assessed to evaluate recurrent or persistent rHPT after PTx in patients with ESRD undergoing hemodialysis and peritoneal dialysis [9, 10]. Patients who are indicated for re-PTx, according to the guidelines, require a detailed investigation to localize the causative PTGs [9, 10]. Investigating these causative PTGs without prediction is inefficient because evaluating the neck, mediastinum, and autografted forearm using various imaging studies can be expensive. Before detailed evaluation using expensive imaging studies, predicting the localization of causative PTGs with simple and cost-efficient methods, such as the Casanova and simplified Casanova tests, may be convenient. However, these methods require avascularization of the autografted forearm for >10 min to compare the intact PTH levels before and after avascularization with a tourniquet or Esmarch [11, 12]. This may cause a great burden to the patients because avascularization with a tourniquet or Esmarch is incredibly painful and can cause nerve damage due to heavy pressure [13]. In this study, we developed and investigated a new, simple, and inexpensive method to predict the localization of causative PTGs in recurrent or persistent rHPT.

## Materials and methods

### Study design

This retrospective cohort study was approved by Nagoya Daini Red Cross Hospital’s Institutional Review Board (Aichi, Japan) (approval number: 1268) and was conducted according to the Declaration of Helsinki. The need for informed consent was waived by Nagoya Daini Red Cross Hospital’s Institutional Review Board because of the retrospective nature of the study.

The intact PTH levels obtained before re-PTx, according to the blood sampling method and the localization diagnosed in the re-PTx, were used for the cutoff levels of the intact PTH ratio (the intact PTH level obtained from the non-autografted forearm before re-PTx/intact PTH level obtained from the autografted forearm before re-PTX). The cutoff levels of the intact PTH ratio were investigated with receiver operating characteristic (ROC) curves to detect the localization of causative PTGs. This study is reported in accordance with the Strengthening the Reporting of Observational Studies in Epidemiology guidelines.

### Participants

In total, 210 patients underwent re-PTx for persistent or recurrent rHPT after total PTx and forearm autograft between January 2000 and July 2019 at our center. Data were collected retrospectively from the patients’ charts in March 2020 and analyzed anonymously.

### Indications for re-PTx for recurrent or persistent rHPT

According to the clinical practice guidelines for the management of chronic kidney disease-mineral and bone disorder, re-PTx for recurrent or persistent rHPT was indicated for patients with intact PTH levels higher than 500 pg/mL, in whom calcimimetics could not be administered due to its adverse effects, and whose swollen autografted PTGs were identified with ultrasonography and magnetic resonance imaging [9].

#### Diagnosis of localization of PTGs in PTx and re-PTx

Localization of PTGs was diagnosed with ultrasonography, computed tomography, and technetium-99m methoxyisobutylisonitrile (^99m^Tc-MIBI) scintigraphy for PTx and re-PTx in the neck or mediastinum [14]. The localization of PTGs in the autografted forearm was diagnosed with ultrasonography and magnetic resonance imaging.

#### PTx and re-PTx procedures in the autografted forearm and neck or mediastinum

For all patients enrolled in this study, total PTx and transcervical thymectomy with forearm autograft were performed during PTx. For the re-PTx in the autografted forearm, en bloc resection of autografted PTGs with surrounding muscle was performed. For re-PTx in the neck, removal of PTGs from the neck incision was performed. For re-PTx in the mediastinum, an open or thoracoscopic approach was used.

#### Definition of successful re-PTx

Successful re-PTx in the neck or mediastinum is defined based on whether the intact PTH level on POD 1 is <180 pg/mL. Accordingly, successful re-PTx in the autografted forearm is defined based on whether the intact PTH level on POD 1 is <60 pg/mL.

Maintaining intact PTH levels of <180 pg/mL in patients undergoing dialysis is recommended in clinical practice for the management of rHPT in patients requiring chronic dialysis because the 1-year survival of such patients with intact PTH levels of <180 pg/mL has been demonstrated to be significantly better than that in patients with intact PTH levels ranging from 180–360 pg/mL [15, 16]. Intact PTH levels of <180 pg/mL on POD 1 after re-PTx could indicate successful re-PTx in the neck or mediastinum because patients whose remnant PTGs in the neck and mediastinum had been removed still have autografted PTGs producing intact PTH in the forearm.

The validity of an intact PTH level of <60 pg/mL on POD 1 for successful total PTx and transcervical thymectomy with forearm autograft was demonstrated in our previous study [17]. After removing the autografted PTGs, PTGs should not remain in the forearm and neck or mediastinum. Because this condition is the same as that on POD 1 in total PTx and transcervical thymectomy with forearm autograft, an intact PTH level of <60 pg/mL on POD 1 after re-PTx in the autografted forearm was defined.

### Blood sampling method

The intact PTH levels were measured from blood samples obtained from the bilateral forearm 5 min after the avascularization in the upper arm of autografted PTGs with the usual rubber band (thickness, 0.6 mm; width, 25 mm; and length, 45 cm) rather than a tourniquet or Esmarch.

### Measurement of intact PTH levels

The intact PTH levels were measured using ST AIA-PACK Intact PTH (Tosoh Corporation, Tokyo, Japan).

### Statistical analyses

Statistical analyses were performed using the t-test or Mann–Whitney U test for continuous variables and Fisher’s exact test for categorical variables. The results are presented as mean (standard deviation) or median (interquartile range). Patients were classified into true positive, true negative, false positive, and false negative. Sensitivity, specificity, positive predictive value, negative predictive value, and accuracy were calculated using the following formulas:

Sensitivity = [True positive / (True positive + False negative)] × 100
Specificity = [True negative / (False positive + True negative)] × 100
Positive predictive value = [True positive / (True positive + False positive)] × 100
Negative predictive value = [True negative / (False negative + True negative)] × 100
Accuracy = (True positive + True negative) / (True positive + False positive + False negative + True negative) × 100.

ROC curve analysis was used for the cutoff value of the intact PTH ratio. All statistical analyses were performed using the Statistical Package for the Social Sciences software, version 23.0 for Windows (IBM Corporation, Armonk, NY, USA). For all analyses, a P-value <0.05 was considered statistically significant.

## Results

### Participants

In total, 210 patients underwent re-PTx for persistent or recurrent rHPT after total PTx and transcervical thymectomy with forearm autograft between January 2000 and July 2019 at our center.

**Fig 1:**
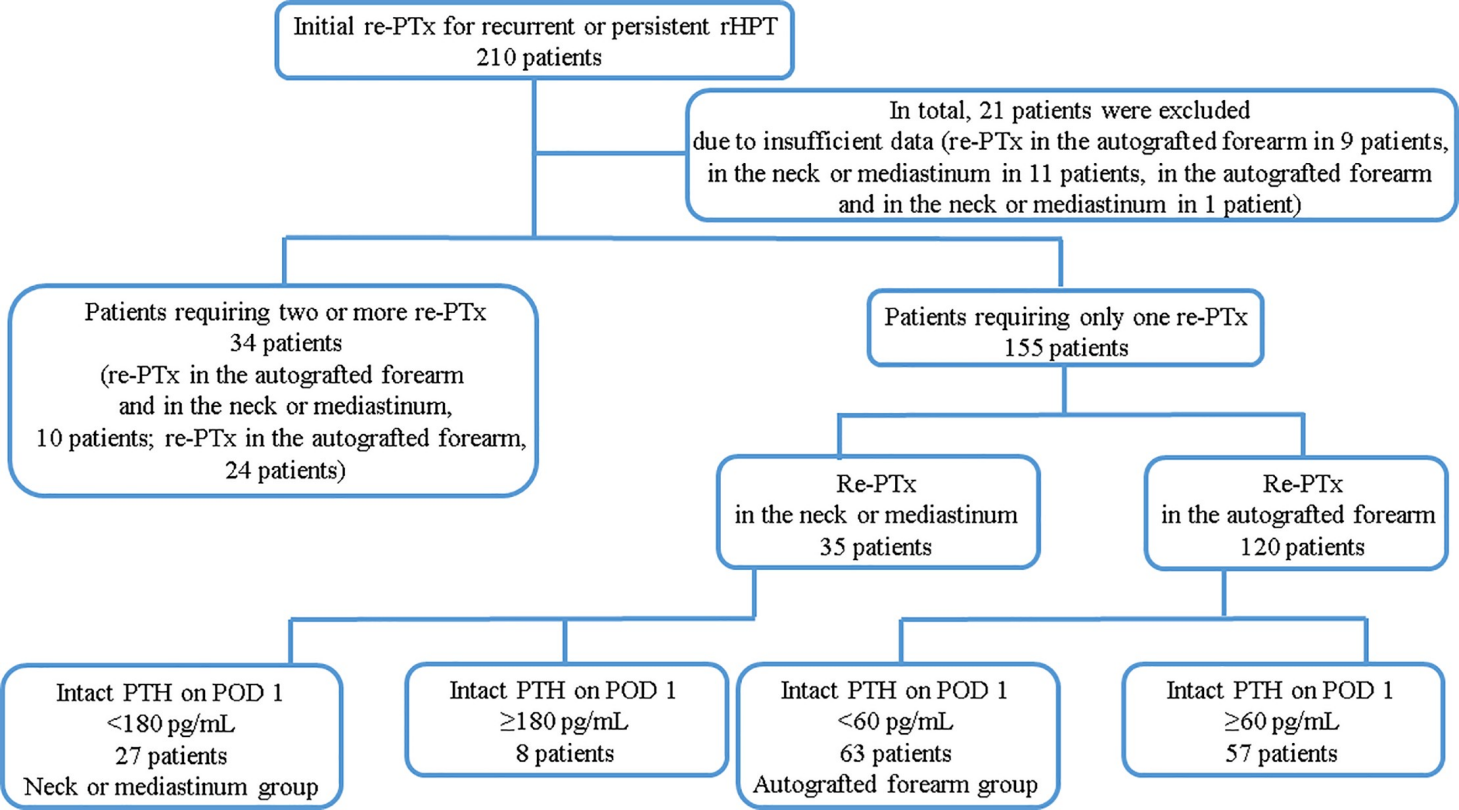
Patient flow chart. PTx, parathyroidectomy; POD 1, postoperative day 1; PTH, parathyroid hormone; rHPT, renal hyperparathyroidism.

Twenty-one patients were excluded because of insufficient data (Fig 1). In nine of the 21 patients, re-PTx for recurrent or persistent rHPT was performed in the autografted forearm; in 11 of the 21 patients, re-PTx was performed in the neck or mediastinum; and in one of the 21 patients, re-PTx was performed in the autografted forearm and the neck or mediastinum. Twenty-one patients were excluded because of lack of data on preoperative intact PTH levels obtained from the forearm in 18 patients and on intact PTH levels on admission or POD 1 in three patients.

In total, 189 patients were included in this study. One hundred and fifty-five of the 189 patients required only one re-PTx. Of those, 35 patients underwent re-PTx in the neck or mediastinum. Of the 35 patients, re-PTx was successful in 27 patients (intact PTH level <180 pg/mL on POD 1, neck or mediastinum group). In total, 120 of 155 patients underwent re-PTx in the autografted forearm. Of those, re-PTx was successful in 63 patients (intact PTH level <60 pg/mL on POD 1, autografted forearm group).

Of the 189 patients, 34 required two or more re-PTx, 10 of the 34 patients required re-PTx in the autografted forearm and the neck or mediastinum, and 24 of the 34 patients required re-PTx in the autografted forearm.

This study examined the data of the patients who were classified into the neck or mediastinum and autografted forearm groups (Fig 1). Patients were followed up every 3–12 months after PTx and re-PTx. The average follow-up period of the patients was 182.0 (interquartile range: 145.0–208.0) months.

### Descriptive data

Table 1 presents the patients’ characteristics classified into the autografted forearm group and the neck or mediastinum group. No significant differences were observed in sex, age at PTx, and hemodialysis vintage before PTx. Table 2 presents the details of the PTx and re-PTx. In the PTx, the number of removed PTGs was significantly higher in the autografted forearm group (P <0.001). In contrast, the intact PTH levels on POD 1 in PTx were significantly higher in the neck or mediastinum group (P <0.001). Regarding re-PTx, the intact PTH level obtained from the autografted forearm was significantly higher in the autografted forearm group (P <0.001), although the intact PTH levels obtained from the non-autografted forearm were similar between the two groups (P = 0.236). The intact PTH level obtained from the non-autografted forearm after re-PTx was significantly higher in the neck or mediastinum group (P = 0.030), although their intact PTH levels were within the normal range (9–80 pg/mL).

**Table 1:** Patient characteristics.

	Autografted forearm group	Neck or mediastinum group	t-test, Mann-Whitney U test, or Fisher’s exact test			
	N = 63	N = 27	P-value	Mean difference	Odds ratio	95% CI
**Sex (male, %)**	32 (50.8%)	13 (48.1%)	> 0.999		0.900	0.365–2.217
**Age at PTx (years, SD)**	56.3 (7.8)	53.6 (8.2)	0.142	1.820		-0.923–6.309
**Age at re-PTx (years, SD)**	62.1 (7.8)	57.1 (8.1)	0.006	1.811		1.453–8.653
**HD vintage before PTx (months, interquartile)**	119.0 (87.5–142.0)	132.0 (92.5–164.5)	0.265	13.0		-12.0–42.0
**The period between PTx and re-PTx (months, interquartile)**	62.0 (43.0–88.5)	35.0 (18.5–60.0)	< 0.001	-27.0		-41.0 –-12.0
**Follow-up period (months, interquartile)**	195.0 (160.5–212.5)	155.0 (100.0–186.5)	0.007	-40.0		-58.0 –-10.0

CI, confidence interval; HD, hemodialysis; SD, standard deviation; PTx, parathyroidectomy.

Results are presented as mean (standard deviation) or median (interquartile range).

**Table 2:** The details of parathyroidectomy and re-parathyroidectomy.

		Autografted forearm group	Neck or mediastinum group	Mann-Whitney U test		
		N = 63	N = 27	P-value	Mean difference	95% CI
**PTx**	**Number of removed parathyroid glands (interquartile)**	4.0 (4.0–4.0)	4.0 (3.0–4.0)	< 0.001	0	-1.0–0
	**Intact PTH levels on admission (pg/mL, interquartile)**	780.0 (580.0–1,100.0)	840.0 (647.0–1,100.0)	0.660	40.0	-160.0–230.0
	**Intact PTH levels on POD 1 (pg/mL, interquartile)**	14.0 (9.0–24.0)	92.0 (53.0–260.0)	< 0.001	74.0	53.0–147.0
	**Serum calcium levels on admission (mg/dL, interquartile)**	10.80 (10.30–11.20)	10.80 (10.30–11.40)	0.944	0.01	-0.40–0.37
	**Serum calcium levels on POD 1 (mg/dL, interquartile)**	8.90 (8.40–9.70)	9.10 (8.60–9.40)	0.465	0.14	-0.30–0.48
	**Serum phosphorus levels on admission (mg/dL, interquartile)**	5.40 (5.00–6.40)	5.70 (4.70–6.20)	0.839	-0.10	-0.60–0.50
	**Serum phosphorus levels on POD 1 (mg/dL, interquartile)**	5.40 (4.80–6.30)	5.80 (4.90–6.20)	0.465	0.20	-0.30–0.80
**Re-PTx**	**Preoperative intact PTH levels obtained from the autografted forearm (pg/mL, interquartile)**	3,850.0 (1,220.0–7,864.0)	463.0 (321.5–1,331.5)	< 0.001	-2,870.0	-4621.0 –-1330.0
	**Preoperative intact PTH level obtained from the non-autografted forearm (pg/mL, interquartile)**	341.0 (238.5–484.5)	354.0 (199.0–780.5)	0.236	76.0	-49.0–240.0
	**Intact PTH levels obtained from the non-autografted forearm on admission (pg/mL, interquartile)**	408.0 (295.5–587.5)	616.0 (399.5–883.0)	0.004	200.0	61.0–341.0
	**Intact PTH levels obtained from the non-autografted forearm on POD 1 (pg/mL, interquartile)**	24.0 (12.00–39.50)	42.0 (12.00–79.50)	0.030	16.0	1.0–35.3
	**Serum calcium levels on admission (mg/dL, interquartile)**	10.20 (9.70–10.70)	10.20 (9.70–11.30)	0.745	0.07	-0.30–0.51
	**Serum calcium levels on POD 1 (mg/dL, interquartile)**	8.90 (8.40–9.70)	8.90 (8.40–9.20)	0.951	0	-0.45–0.34
	**Serum phosphorus levels on admission (mg/dL, interquartile)**	5.60 (4.70–6.00)	5.00 (4.50–6.00)	0.189	-0.40	-0.90–0.20
	**Serum phosphorus levels on POD 1 (mg/dL, interquartile)**	5.40 (4.60–6.30)	5.10 (4.90–5.90)	0.874	0	-0.60–0.50

PTx, parathyroidectomy; POD 1, post-operative day 1; PTH, parathyroid hormone.

Results are presented as median (interquartile range).

### Intact PTH level changes before and after re-PTx

**Fig 2:**
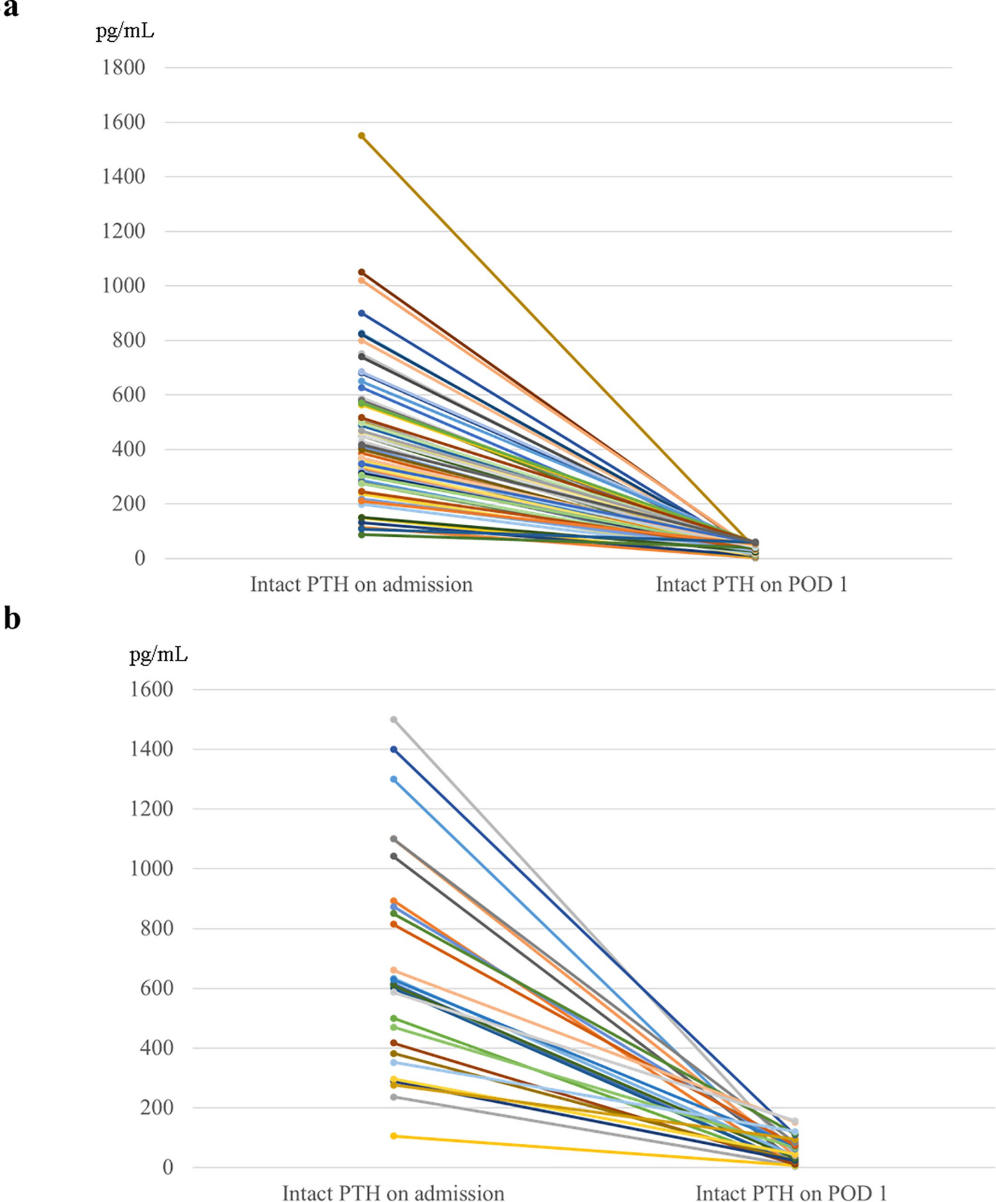
Intact PTH levels on admission and POD 1 in re-parathyroidectomy for the recurrent or persistent renal hyperparathyroidism in the autografted forearm group (a) and the neck or mediastinum group (b). PTH, parathyroid hormone; POD 1, postoperative day 1.

In the autografted forearm and neck or mediastinum groups, the intact PTH levels obtained from the non-autografted forearm decreased after re-PTx (Fig 2A and 2B).

### Intact PTH ratios of the autografted forearm and neck or mediastinum groups

**Fig 3:**
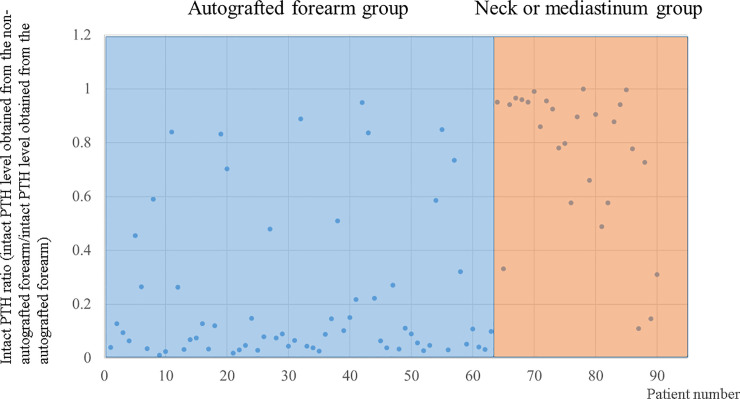
Intact PTH ratios in the autografted forearm group and the neck or mediastinum group. PTH, parathyroid hormone.

**Fig 4:**
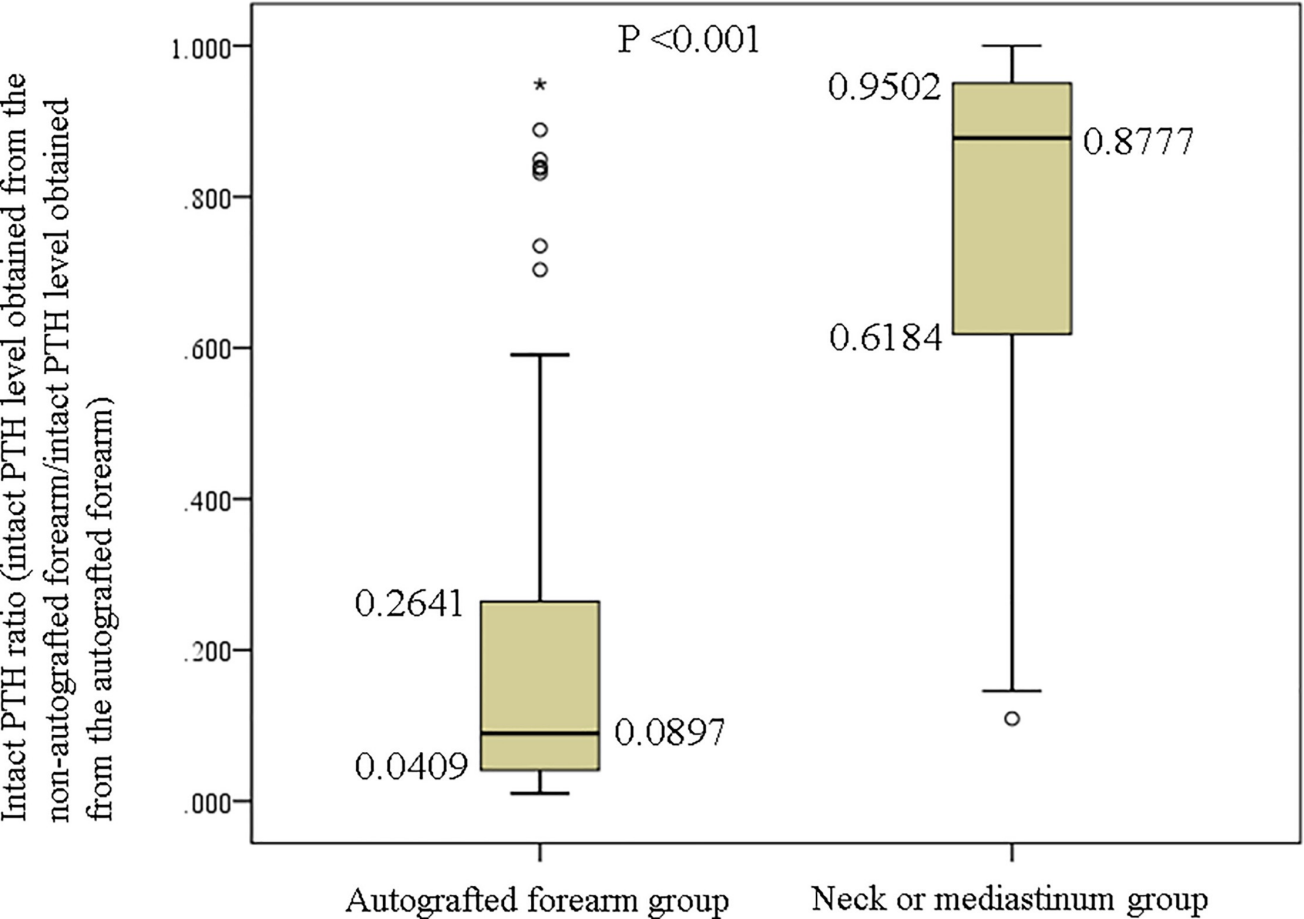
Comparison of intact PTH ratios in the autografted forearm group and the neck or mediastinum group. PTH, parathyroid hormone.

The intact PTH ratios of the autografted forearm and neck or mediastinum groups are plotted in Fig 3. The intact PTH ratios of the autografted forearm group concentrated in the lower ratio, whereas those of the neck or mediastinum group concentrated in the higher ratio. The intact PTH ratios were significantly lower in the autografted forearm group than in the neck or mediastinum group (median 0.0897 vs. 0.8777; P <0.001) (Fig 4).

### ROC analysis for the cutoff value to identify recurrence or persistence in the autografted forearm

**Fig 5:**
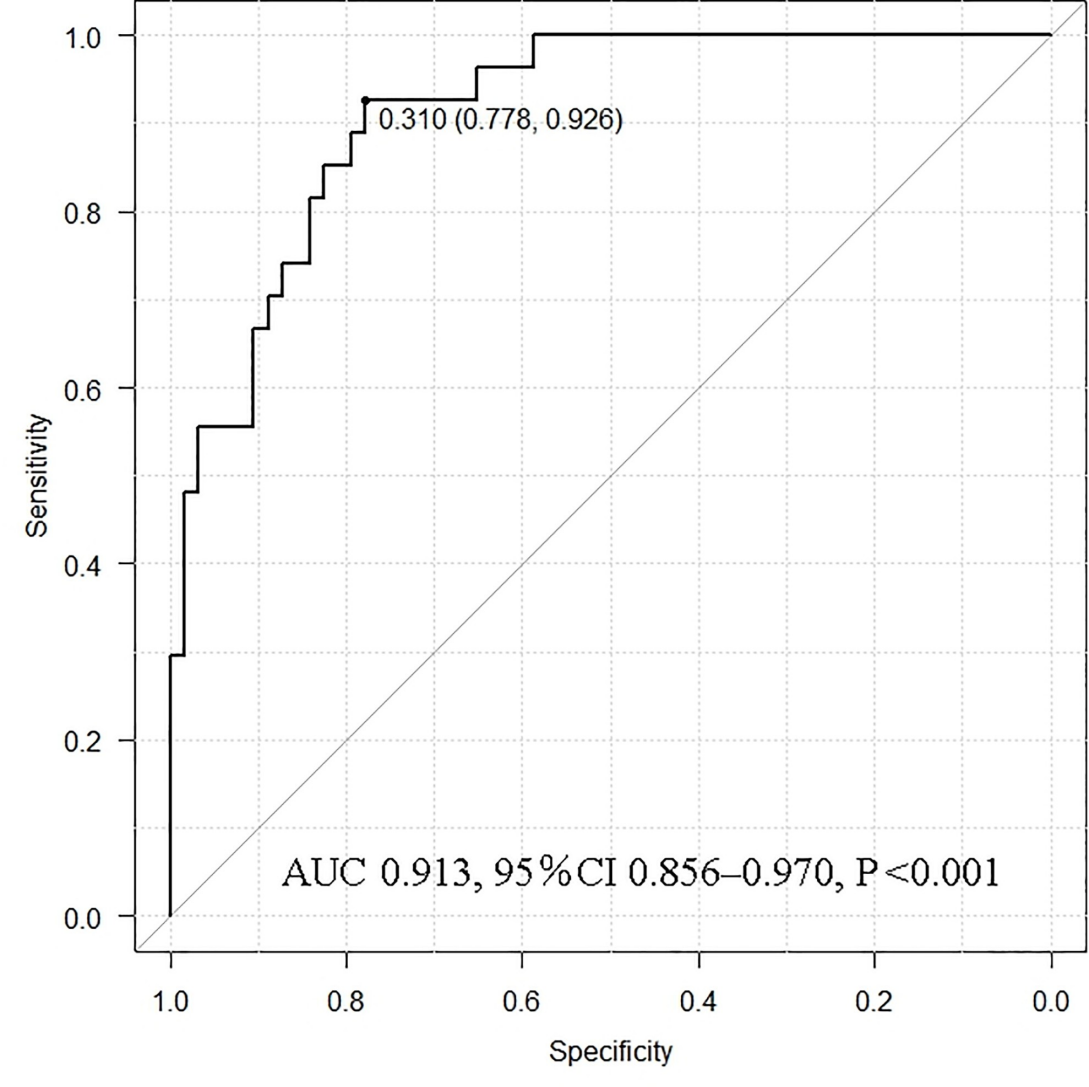
Receiver operating characteristic curve analysis for the cutoff value of recurrence or persistence in the autografted forearm. AUC, area under the curve; CI, confidence interval.

ROC curve analysis showed the best sensitivity and specificity at 0.310 for an intact PTH ratio of 0.913 (95% confidence interval [CI]: 0.856–0.970; P <0.001) in the area under the curve (AUC) (Fig 5). The sensitivity, specificity, positive predictive value, negative predictive value, and accuracy calculated from S1 Table were 77.7%, 88.9%, 94.2%, 63.2%, and 81.1%, respectively.

### ROC analysis for the cutoff value to identify recurrence or persistence in the neck or mediastinum

**Fig 6:**
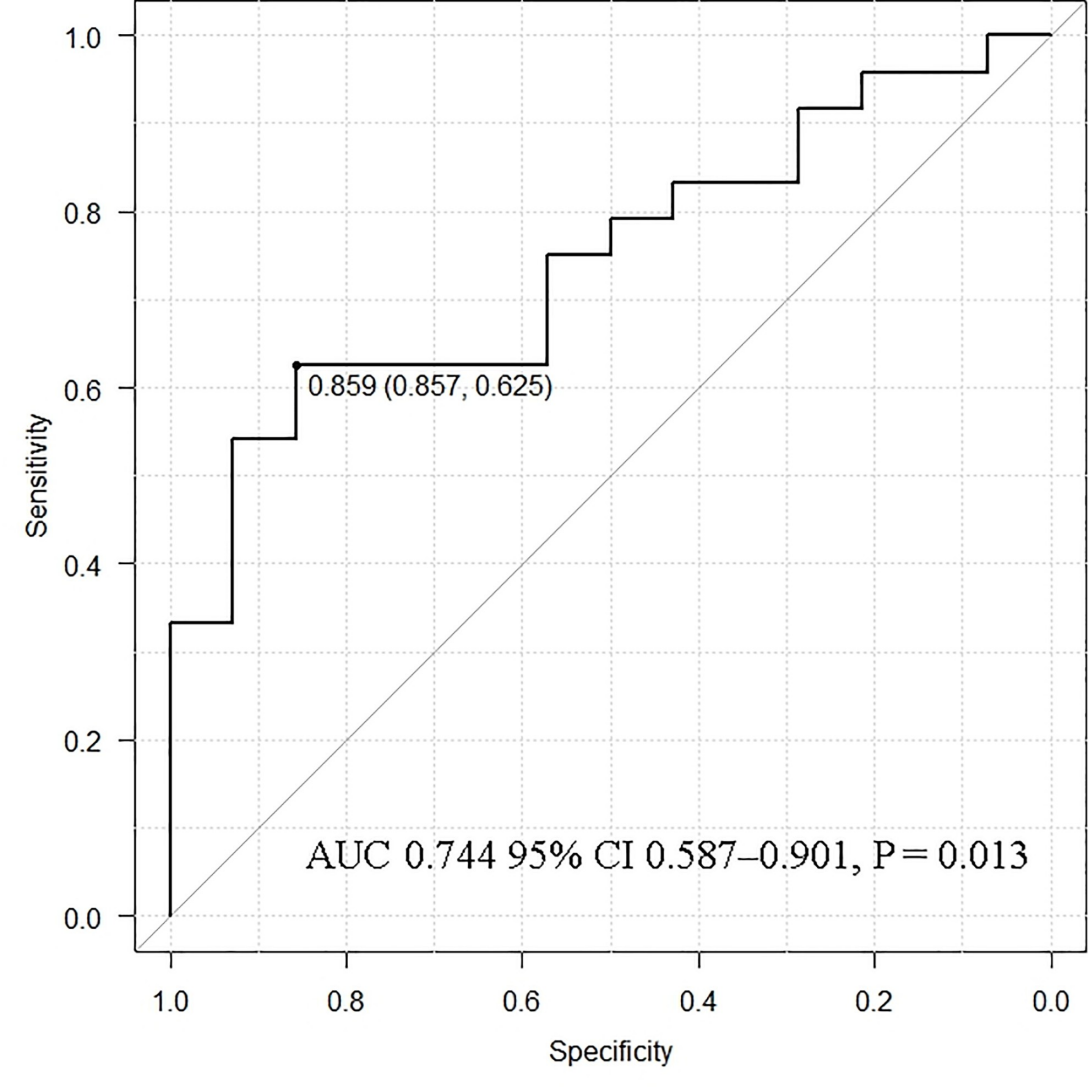
Receiver operating characteristic curve analysis for the cutoff value of recurrence or persistence in the neck or mediastinum. AUC, area under the curve; CI, confidence interval.

ROC curve analysis was performed for an intact PTH ratio of >0.310 to identify recurrence or persistence in the neck or mediastinum. It showed the best sensitivity and specificity at 0.859 for an intact PTH ratio of 0.744 (95% CI 0.587–0.901; P = 0.013) in the AUC (Fig 6). The sensitivity, specificity, positive predictive value, negative predictive value, and accuracy evaluated for all intact PTH ratios in S2 Table were 83.3%, 83.3%, 55.6%, 95.2%, and 83.3%, respectively.

### Recommended algorithm for the diagnostic localization of causative PTGs for recurrent or persistent rHPT

Intact PTH ratios of <0.310 and >0.859 imply that the autografted forearm and the neck or mediastinum should be evaluated, respectively. An intact PTH ratio between 0.310 and 0.859 implies that both the autografted forearm and the neck or mediastinum should be evaluated (Table 3).

**Table 3:** Algorithm for the localization of causative parathyroid glands for recurrent or persistent renal hyperparathyroidism after total parathyroidectomy with forearm autograft.

Intact PTH ratio (intact PTH level obtained from the non-autografted forearm before re-parathyroidectomy/intact PTH level obtained from the autografted forearm before re-parathyroidectomy)	<0.310	Examined in the autografted forearm
	0.310–0.859	Examined both in the autografted forearm and the neck or mediastinum
	>0.859	Examined in the neck or mediastinum

## Discussion

In this study, intact PTH ratios of <0.310 and >0.859 were found to be useful for predicting the localization of causative PTGs for recurrent or persistent rHPT before detailed imaging studies. Recurrent or persistent rHPT may occur because of insufficient PTx or continuous stimulation under a chronic kidney disease condition [1, 18, 19]. Hypocalcemia due to vitamin D deficiency and hyperphosphatemia due to decreased phosphorus excretion in the urine stimulate the remnant PTGs and cause enlargement. To prevent recurrent or persistent rHPT, total PTx and transcervical thymectomy are recommended [20, 21]. The need for an autograft remains controversial [22–25]. In this study, total PTx and transcervical thymectomy with forearm autograft were performed for PTx. For recurrent or persistent rHPT, re-PTx is necessary if rHPT is refractory to medications [9]. Calcimimetics, activated vitamin D, and calcium tablets are often administered to treat rHPT [26, 27]. Calcimimetics have become the mainstay of treatment for rHPT over the last decade [28]. However, these treatments are not effective in advanced rHPT [29]. Adverse effects due to calcimimetics, such as nausea, may cause withdrawal [9, 30]. While patients were followed up at our hospital, intact PTH levels were measured to exclude recurrent or persistent rHPT [9, 10]. Moreover, performing various imaging studies for the localization of causative PTGs without predicting when intact PTH levels would increase can be costly for patients; thus, a simple method to predict the localization before the imaging studies is necessary. Recurrent or persistent rHPT may occur in the autografted forearm or the neck or mediastinum. Evaluating both areas simultaneously is difficult because appropriate modalities for the localization differ according to the location. For the localization of recurrent or persistent rHPT in the forearm, magnetic resonance imaging, ^99m^Tc -MIBI scintigraphy, and ultrasonography are appropriate [5–8], whereas in the neck or mediastinum, computed tomography, ^99m^Tc-MIBI scintigraphy, and single-photon emission computed tomography are suitable [2–5]. The Casanova or simplified Casanova test was developed to make informed decisions on the areas for evaluation. However, the number of enrolled patients in the previous studies was limited, and the duration of avascularization in the tests was >10 min [11, 12]. Avascularization with a tourniquet or the use of Esmarch for the Casanova or simplified Casanova test is a heavy burden on the patients. Therefore, we developed a simple method to predict the localization of causative PTGs for rHPT before detailed imaging studies [11, 12]. In our method, the duration of avascularization is only 5 min, and a normal rubber band used for blood sampling is used for avascularization. These simple techniques in this newly developed method can reduce patient burden and lower the laboratory technician’s time and efforts.

In this study, it was important to localize recurrent or persistent PTGs. Patients who did not require additional re-PTx were involved in the evaluation for the appropriate intact PTH ratio. Patients were classified into the autografted forearm group and the neck or mediastinum group according to the location of the recurrent or persistent PTGs. Between these two groups, no significant differences were observed in sex, age at PTx, and hemodialysis vintage before PTx (Table 1). In the PTx, the number of removed PTGs was significantly higher in the autografted forearm group. In contrast, the intact PTH levels on POD 1 in PTx were significantly lower in the autografted forearm group (Table 2). These results demonstrate that in the autografted forearm group, the PTGs had been successfully removed at PTx, and recurrent rHPT occurred only in the autografted forearm. The significantly shorter periods between PTx and re-PTx and the significantly higher intact PTH level on POD 1 after PTx in the neck or mediastinum group imply that remnant PTGs might cause persistent rHPT and lead to earlier re-PTx (Tables 1 and 2) [20, 31]. Regarding re-PTx, the intact PTH level obtained from the non-autografted forearm after re-PTx was significantly higher in the neck or mediastinum group than in the autografted forearm group (Table 2). This implies that in the neck or mediastinum group, the PTGs in the autografted forearm remained and still produced intact PTH after removing PTGs in the neck or mediastinum at re-PTx. On the contrary, in the autografted forearm group, the PTGs did not remain in the forearm and neck or mediastinum after re-PTx and presented significantly lower intact PTH levels. In the patients who underwent successful re-PTx in the autografted forearm and neck or mediastinum groups, intact PTH levels on POD 1 decreased adequately compared to the levels on admission (Fig 2A and 2B). The appropriate cutoff level of intact PTH ratio for the localization of causative PTGs in recurrent or persistent rHPT was investigated in the autografted forearm group and neck or mediastinum group (Fig 3). In re-PTx, the preoperative intact PTH level obtained from the autografted forearm was significantly higher in the autografted forearm group, although preoperative intact PTH levels obtained from the non-autografted forearm were similar in both groups (Table 2). The results led to a significant difference in the intact PTH ratio (Fig 4). The cutoff values of the intact PTH ratio of <0.310 for the causative PTGs in the autografted forearm and >0.859 for the causative PTGs in the neck or mediastinum by the ROC curve analysis demonstrated good accuracies of 81.1% and 83.3%, respectively, in the 90 patients who underwent successful re-PTx. (Figs 5 and 6, S1 and S2 Tables). This result implies that the autografted forearm should be evaluated when the intact PTH ratio is <0.310, and the neck or mediastinum should be evaluated when the intact PTH ratio is >0.859. However, an intact PTH ratio between 0.321 and 0.849 cannot predict the location of the causative PTGs. Both the autografted forearm and neck or mediastinum should be evaluated in such cases. These results led to the recommended algorithm (Table 3).

The limitation of this study was its retrospective nature. Therefore, future prospective randomized control studies on the intact PTH ratio to predict causative PTGs should be conducted.

In conclusion, an intact PTH ratio is useful for predicting the localization of causative PTGs for recurrent or persistent rHPT without detailed imaging studies. The recommended algorithm will be helpful for the management of rHPT in clinical practice.

## Supporting information

S1 Table Contingency table of the intact PTH ratio. (DOCX)

S2 Table Contingency table of the intact PTH ratio. (DOCX)

S1 Checklist (DOC)

## References

[1] Tominaga Y, Katayama A, Sato T, Matsuoka S, Goto N, Haba T, et al. Re-operation is frequently required when parathyroid glands remain after initial parathyroidectomy for advanced secondary hyperparathyroidism in uraemic patients. Nephrol Dial Transplant. 2003;18: 65–70. 10.1093/ndt/gfg1017 12771305

[2] Karipineni F, Sahli Z, Somervell H, Mathur A, Prescott JD, Tufano RP, et al. Are preoperative sestamibi scans useful for identifying ectopic parathyroid glands in patients with expected multigland parathyroid disease? Surgery. 2018;163: 35–41. 10.1016/j.surg.2017.07.035 29154082

[3] de Andrade JS, Mangussi-Gomes JP, da Rocha LA, Ohe MN, Rosano M, das Neves MC, et al. Localization of ectopic and supernumerary parathyroid glands in patients with secondary and tertiary hyperparathyroidism: surgical description and correlation with preoperative ultrasonography and Tc99m-Sestamibi scintigraphy. Braz J Otorhinolaryngol. 2014;80: 29–34. 10.5935/1808-8694.20140008 24626889 PMC9443960

[4] Taïeb D, Ureña-Torres P, Zanotti-Fregonara P, Rubello D, Ferretti A, Henter I, et al. Parathyroid scintigraphy in renal hyperparathyroidism: the added diagnostic value of SPECT and SPECT/CT. Clin Nucl Med. 2013;38: 630–635. 10.1097/RLU.0b013e31829af5bf 23751837 PMC4300197

[5] Chou FF, Lee CH, Chen HY, Chen JB, Hsu KT, Sheen-Chen SM. Persistent and recurrent hyperparathyroidism after total parathyroidectomy with autotransplantation. Ann Surg. 2002;235: 99–104. 10.1097/00000658-200201000-00013 11753048 PMC1422401

[6] Itoh K, Ishizuka R. Tc-99m-MIBI scintigraphy for recurrent hyperparathyroidism after total parathyroidectomy with autograft. Ann Nucl Med. 2003;17: 315–320. 10.1007/BF02988528 12932116

[7] Hergan K, Neyer U, Doringer W, Mündle M. MR imaging in graft-dependent recurrent hyperparathyroidism after parathyroidectomy and autotransplantation. J Magn Reson Imaging. 1995;5: 541–544. 10.1002/jmri.1880050511 8574038

[8] Tominaga Y. Surgical management of secondary and tertiary hyperparathyroidism. In: Randolph GW, editor. Surgery of the Thyroid and Parathyroid Glands. 2nd ed. Elsevier Saunders; 2012. pp. 639–647.

[9] Fukagawa M, Yokoyama K, Koiwa F, Taniguchi M, Shoji T, Kazama JJ, et al. Clinical practice guideline for the management of chronic kidney disease-mineral and bone disorder. Ther Apher Dial. 2013;17: 247–288. 10.1111/1744-9987.12058 23735142

[10] Kidney Disease: Improving Global Outcomes (KDIGO) CKD-MBD Update Work Group KDIGO 2017 Clinical practice guideline update for the diagnosis, evaluation, prevention, and treatment of chronic kidney disease-mineral and bone disorder (CKD-MBD). Kidney Int Suppl. 2017;7: 1–59. 10.1016/j.kisu.2017.04.001 PMC6340919 30675420

[11] Casanova D, Sarfati E, De Francisco A, Amado JA, Arias M, Dubost C. Secondary hyperparathyroidism: diagnosis of site of recurrence. World J Surg. 1991;15: 546–550. 10.1007/BF01675660 1891942

[12] Schlosser K, Sitter H, Rothmund M, Zielke A. Assessing the site of recurrence in patients with secondary hyperparathyroidism by a simplified Casanova autograftectomy test. World J Surg. 2004;28: 583–588. 10.1007/s00268-004-7321-8 15366749

[13] Masri BA, Eisen A, Duncan CP, McEwen JA. Tourniquet-induced nerve compression injuries are caused by high-pressure levels and gradients—a review of the evidence to guide safe surgical, pre-hospital, and blood flow restriction usage. BMC Biomed Eng. 2020;2: 7. 10.1186/s42490-020-00041-5 32903342 PMC7422508

[14] Hiramitsu T, Tomosugi T, Okada M, Futamura K, Tsujita M, Goto N, et al. Pre-operative localisation of the parathyroid glands in secondary hyperparathyroidism: a retrospective cohort study. Sci Rep. 2019;9: 14634. 10.1038/s41598-019-51265-y 31602011 PMC6787184

[15] Guideline Working Group, Japanese Society for Dialysis Therapy. Clinical practice guideline for the management of secondary hyperparathyroidism in chronic dialysis patients. Ther Apher Dial. 2008;12: 514–525. 10.1111/j.1744-9987.2008.00648.x 19140852

[16] Nakai S, Akiba T, Kazama J, Yokoyama K, Fukagawa M, Tominaga Y, et al. Effects of serum calcium, phosphorous, and intact parathyroid hormone levels on survival in chronic hemodialysis patients in Japan. Ther Apher Dial. 2008;12: 49–54. 10.1111/j.1744-9987.2007.00540.x 18257812

[17] Hiramitsu T, Tominaga Y, Okada M, Yamamoto T, Kobayashi T. A retrospective study of the impact of intraoperative intact parathyroid hormone monitoring during total parathyroidectomy for secondary hyperparathyroidism: STARD study. Medicine (Baltimore). 2015;94: e1213. 10.1097/MD.0000000000001213 26200645 PMC4603015

[18] Hibi Y, Tominaga Y, Sato T, Katayama A, Haba T, Uchida K, et al. Reoperation for renal hyperparathyroidism. World J Surg. 2002;26: 1301–1307. 10.1007/s00268-002-6731-8 12205559

[19] Dotzenrath C, Cupisti K, Goretzki P, Mondry A, Vossough A, Grabensee B, et al. Operative treatment of renal autonomous hyperparathyroidism: cause of persistent or recurrent disease in 304 patients. Langenbecks Arch Surg. 2003;387: 348–354. 10.1007/s00423-002-0322-x 12536330

[20] Schneider R, Bartsch DK, Schlosser K. Relevance of bilateral cervical thymectomy in patients with renal hyperparathyroidism: analysis of 161 patients undergoing reoperative parathyroidectomy. World J Surg. 2013;37: 2155–2161. 10.1007/s00268-013-2091-9 23674256

[21] Uludag M, Yetkin G, Citgez B, Ozguven BY, Cengiz AN, Ozsahin H, et al. The role of cervical thymectomy in surgical treatment of secondary hyperparathyroidism. Bratisl Lek Listy. 2011;112: 385–389. 21744733

[22] Schlosser K, Bartsch DK, Diener MK, Seiler CM, Bruckner T, Nies C, et al. Total parathyroidectomy with routine thymectomy and autotransplantation versus total parathyroidectomy alone for secondary hyperparathyroidism: results of a nonconfirmatory multicenter prospective randomized controlled pilot trial. Ann Surg. 2016;264: 745–753. 10.1097/SLA.0000000000001875 27741007

[23] Puccini M, Carpi A, Cupisti A, Caprioli R, Iacconi P, Barsotti M, et al. Total parathyroidectomy without autotransplantation for the treatment of secondary hyperparathyroidism associated with chronic kidney disease: clinical and laboratory long-term follow-up. Biomed Pharmacother. 2010;64: 359–362. 10.1016/j.biopha.2009.06.006 20435429

[24] Stracke S, Jehle PM, Sturm D, Schoenberg MH, Widmaier U, Beger HG, et al. Clinical course after total parathyroidectomy without autotransplantation in patients with end-stage renal failure. Am J Kidney Dis. 1999;33: 304–311. 10.1016/s0272-6386(99)70305-7 10023643

[25] Lorenz K, Bartsch DK, Sancho JJ, Guigard S, Triponez F. Surgical management of secondary hyperparathyroidism in chronic kidney disease—a consensus report of the European Society of Endocrine Surgeons. Langenbecks Arch Surg. 2015;400: 907–927. 10.1007/s00423-015-1344-5 26429790

[26] Tominaga Y, Matsuoka S, Uno N. Surgical and medical treatment of secondary hyperparathyroidism in patients on continuous dialysis. World J Surg. 2009;33; 2335–2342. 10.1007/s00268-009-9943-3 19247704

[27] Zitt E, Rix M, Ureña Torres P, Fouque D, Jacobson SH, Pétavy F, et al. Effectiveness of cinacalcet in patients with recurrent/persistent secondary hyperparathyroidism following parathyroidectomy: results of the ECHO study. Nephrol Dial Transplant. 2011;26: 1956–1961. 10.1093/ndt/gfq641 20947534

[28] van der Plas WY, Engelsman AF, Umakanthan M, Mather A, Sidhu SB, Delbridge LW, et al. Treatment strategy of end-stage renal disease-related hyperparathyroidism before, during, and after the era of calcimimetics. Surgery. 2019;165: 135–141. 10.1016/j.surg.2018.04.092 30413324

[29] Tominaga Y, Matsuoka S, Uno N, Sato T. Parathyroidectomy for secondary hyperparathyroidism in the era of calcimimetics. Ther Apher Dial. 2008;12 Suppl 1: S21–26. 10.1111/j.1744-9987.2008.00627.x 19032523

[30] Block GA, Martin KJ, De Francisco AL, Turner SA, Avram MM, Suranyi MG, et al. Cinacalcet for secondary hyperparathyroidism in patients receiving hemodialysis. N Engl J Med. 2004;350: 1516–1525. 10.1056/NEJMoa031633 15071126

[31] Numano M, Tominaga Y, Uchida K, Orihara A, Tanaka Y, Takagi H. Surgical significance of supernumerary parathyroid glands in renal hyperparathyroidism. World J Surg. 1998;22: 1098–1103. 10.1007/s002689900524 9747174

