# Supplementary material for: Intact parathyroid hormone levels localize causative glands in persistent or recurrent renal hyperparathyroidism: A retrospective cohort study
Source: PLoS One. 2021 Apr 1;16(4):e0248366. doi: 10.1371/journal.pone.0248366 (PMC8016254; doi:10.1371/journal.pone.0248366)
Supplement: S1 Table — (DOCX) [file pone.0248366.s001.docx]

| S1 Table. Contingency table of the intact PTH ratio | | | | |
| --- | --- | --- | --- | --- |
|  |  |  | Recurrence or persistence in the autografted forearm | |
|  |  |  | Positive | Negative |
|  | Intact PTH ratio <0.310 | Positive | TP: 49 | FP: 3 |
|  |  | Negative | FN: 14 | TN: 24 |

FN, false negative; FP, false positive; PTH, parathyroid hormone; TN, true negative; TP, true positive
