# Supplementary material for: Intact parathyroid hormone levels localize causative glands in persistent or recurrent renal hyperparathyroidism: A retrospective cohort study
Source: PLoS One. 2021 Apr 1;16(4):e0248366. doi: 10.1371/journal.pone.0248366 (PMC8016254; doi:10.1371/journal.pone.0248366)
Supplement: S2 Table — (DOCX) [file pone.0248366.s002.docx]

| S2 Table. Contingency table of the intact PTH ratio | | | | |
| --- | --- | --- | --- | --- |
|  |  |  | Recurrence or persistence in the neck or mediastinum | |
|  |  |  |  |  |
|  |  |  | Positive | Negative |
|  | Intact PTH ratio >0.859 | Positive | TP: 15 | FP: 12 |
|  |  | Negative | FN: 3 | TN: 60 |

FN, false negative; FP, false positive; PTH, parathyroid hormone; TN, true negative; TP, true positive
